# A review of structural brain abnormalities in Pallister‐Killian syndrome

**DOI:** 10.1002/mgg3.351

**Published:** 2017-12-09

**Authors:** Cathryn Poulton, Gareth Baynam, Clarissa Yates, Hamid Alinejad‐Rokny, Simon Williams, Helen Wright, Karen J. Woodward, Soruba Sivamoorthy, Joanne Peverall, Peter Shipman, David Ravine, John Beilby, Julian Ik‐Tsen Heng

**Affiliations:** ^1^ Department of Neurology Princess Margaret Hospital Subiaco WA Australia; ^2^ Telethon Kids Institute University of Western Australia Perth WA Australia; ^3^ Genetic Services of Western Australia Perth WA Australia; ^4^ Office of Population Health Genomics Public Health and Clinical Services Division Department of Health Government of Western Australia Perth WA Australia; ^5^ School of Paediatrics and Child Health University of Western Australia Perth WA Australia; ^6^ Institute for Immunology and Infectious Diseases Murdoch University Perth WA Australia; ^7^ Western Australian Register of Developmental Anomalies Perth WA Australia; ^8^ Spatial Sciences, Science and Engineering Curtin University Crawley WA Australia; ^9^ Centre for Medical Research the University of Western Australia Nedlands WA Australia; ^10^ The Harry Perkins Institute of Medical Research QEII Medical Centre Nedlands WA Australia; ^11^ Department of Paediatrics Princess Margaret Hospital Subiaco WA Australia; ^12^ PathWest Laboratory Medicine WA Nedlands WA Australia; ^13^ School of Biomedical Sciences University of Western Australia Perth WA Australia; ^14^ Department of Radiology Princess Margaret Hospital Subiaco WA Australia; ^15^ Curtin Health Innovation Research Institute and Sarich Neuroscience Institute Curtin University Crawley WA Australia

**Keywords:** corpus callosum, Pallister‐Killian syndrome, polymicrogyria, structural brain disorder

## Abstract

**Background:**

Pallister‐Killian syndrome (PKS) is a rare multisystem developmental syndrome usually caused by mosaic tetrasomy of chromosome 12p that is known to be associated with neurological defects.

**Methods:**

We describe two patients with PKS, one of whom has bilateral perisylvian polymicrogyria (PMG), the other with macrocephaly, enlarged lateral ventricles and hypogenesis of the corpus callosum. We have also summarized the current literature describing brain abnormalities in PKS.

**Results:**

We reviewed available cases with intracranial scans (*n* = 93) and found a strong association between PKS and structural brain abnormalities (77.41%; 72/93). Notably, ventricular abnormalities (45.83%; 33/72), abnormalities of the corpus callosum (25.00%; 18/72) and cerebral atrophy (29.17%; 21/72) were the most frequently reported, while macrocephaly (12.5%; 9/72) and PMG (4.17%; 3/72) were less frequent. To further understand how 12p genes might be relevant to brain development, we identified 63 genes which are enriched in the nervous system. These genes display distinct temporal as well as region‐specific expression in the brain, suggesting specific roles in neurodevelopment and disease. Finally, we utilized these data to define minimal critical regions on 12p and their constituent genes associated with atrophy, abnormalities of the corpus callosum, and macrocephaly in PKS.

**Conclusion:**

Our study reinforces the association between brain abnormalities and PKS, and documents a diverse neurogenetic basis for structural brain abnormalities and impaired function in children diagnosed with this rare disorder.

## 1 INTRODUCTION

Pallister‐Killian syndrome (PKS) is a rare, sporadic disorder caused by mosaic tetrasomy 12p and characterized by a supernumerary isochromosome, comprising two short arms of chromosome 12 (OMIM# 601803) (Izumi & Krantz, 2014; Srinivasan & Wright, 2014; Wenger, Boone, & Steele, 1990). Rarely, individuals may have partial or complete duplications of 12p and present with a PKS phenotype (Izumi et al., 2012). Individuals with PKS display distinct facial anomalies (prominent forehead with spared temporal hair, broad nasal bridge, hypertelorism, wide mouth), variable developmental delay and intellectual impairment, hypotonia, seizures, pigmentary skin differences, diaphragmatic hernia, congenital heart defects, and other systemic abnormalities (Izumi et al., 2012; Pallister et al., 1977). Presently, neurological impairments in PKS are anecdotally documented, yet a quantitative assessment of the spectrum of brain abnormalities which could be informative for prognosticating neurodevelopmental outcomes. Here, we describe two probands with PKS with accompanying structural brain abnormalities. We present our findings together with a literature survey of brain abnormalities reported for PKS. We highlight minimal critical regions on 12p and their candidate genes relevant to brain development and disease. Given the mosaic nature of PKS, our findings also serve to raise the index of suspicion for assessment of this syndrome in children with structural brain abnormalities, and the attendant implications for testing.

## 2 METHODS

Standard cytogenetic protocols were used to culture amniocytes, peripheral blood lymphocytes and skin fibroblasts and to perform conventional G‐banded chromosome analysis. Fluorescence in situ hybridization (FISH) was performed using subtelomeric probes for chromosome 12p (RP11‐283I3) and 12q (RP11‐46H11). Probes were labeled with SpectrumOrange and SpectrumGreen (Abbott Molecular, USA) and FISH signals were examined using the Applied Imaging CytoVision image capture system (Leica Biosystems, Germany). DNA was extracted from peripheral blood and skin fibroblasts from proband two using standard methods. Chromosomal microarray was performed using an Infinium^®^ HumanCytoSNP‐12 BeadChip assay according to manufacturer's instructions (Illumina, San Diego, CA, USA) and data were analyzed using KaryoStudio software. Magnetic Resonance Imaging (MRI) data were generated using a Siemens Sonata 1.5 tesla scanner. Parents of both probands provided informed consent for this study.

## 3 RESULTS

**Figure 1:**
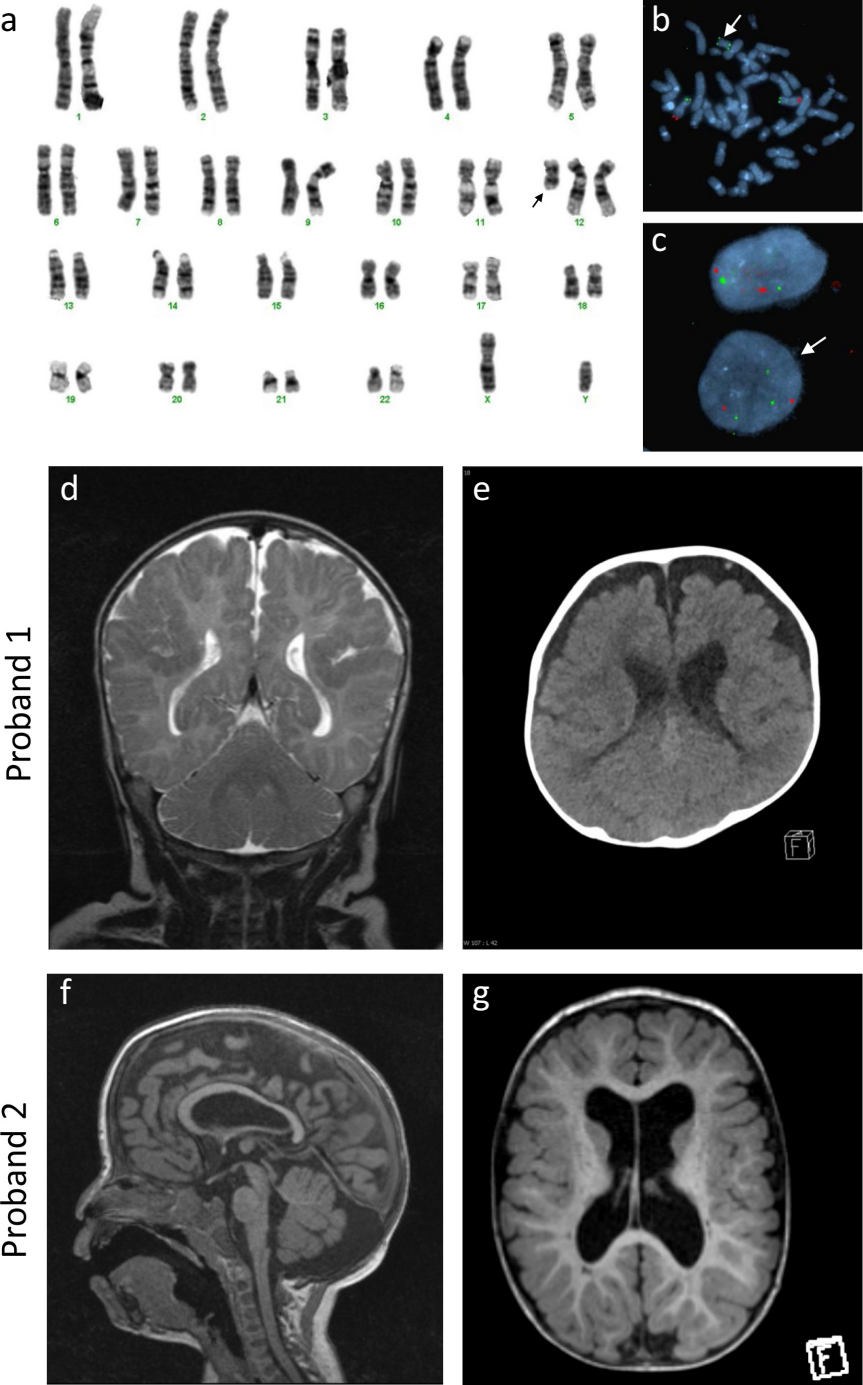
Pallister‐Killian syndrome with structural brain abnormalities. (a) G‐banded chromosome analysis from cultured amniocytes from Proband 1 showed a male karyotype with a supernumerary marker chromosome (shown by the arrow). (b) Metaphase FISH analysis of cultured amniocytes from Proband 1 using subtelomeric probes for chromosome 12p13.33 (RP11‐283I3) (green signal) and 12q24.33 (RP11‐46H11) (red signal) showed the supernumerary marker chromosome with two green signals and indicated isochromosome 12p. (c) Interphase FISH analysis of skin fibroblasts from Proband 2 indicated mosaic trisomy 12p with the presence of three green signals for 12p13.33 (RP11‐283I3) and two red signals for 12q24.33 (RP11‐46H11) in approximately 14% of cells (shown by the arrow). (d–e) MRI and CT scans, respectively, reveal bilateral polymicrogyria in Proband 1. (f–g) MRI representation of thin corpus callosum and a prominent cisterna magna as well as enlarged lateral ventricles in Proband 2

The parents of proband 1 are healthy, nonconsanguinous and of Maori descent. This was the 10th pregnancy with three previous miscarriages, three medically terminated pregnancies and two previous live births with a different partner. Antenatal care was commenced at 28 weeks and was subsequently complicated by polyhydramnios requiring two amnioreductions. Spontaneous preterm labor resulted in a live male born delivered at 33 weeks and 6 days of gestation; the first live birth of this partnership. Delivery was uncomplicated and he required some brief support at birth (Intermittent positive pressure ventilation for <1 min) with APGARs of 6 and 7 at 1 and 5 min, respectively. His birth weight was 2,355 g (56th percentile) and head circumference was 33 cm (79th percentile). The diagnosis of Pallister‐Killian syndrome was made on samples ascertained from the amniotic fluid and results were available on day 2 of life. G‐banded chromosome analysis (400 band resolution) of the amniotic fluid culture revealed a supernumerary marker chromosome (Figure 1a). Fluorescence in situ hybridization (FISH) confirmed this marker was isochromosome i(12p) Figure 1b). Fifteen metaphase cells and 50 interphase nuclei were counted with no evidence of mosaicism. The karyotype was 47,XY,+i(12)(p10) with tetrasomy for the short arm of chromosome 12 (12p).

The infant was noted at birth to have many characteristic features of PKS including coarse facial features, depressed nasal bridge, hearing loss, malalignment of 1st and 2nd toes, contractures of the wrist, some blaschkoid hypopigmented areas of skin and an umbilical hernia. He was hypotonic and had a high‐arched palate. Cardiac and ophthalmic examination was normal.

Proband 1 had a complicated neonatal course requiring intubation and surfactant administration shortly after birth and a prolonged period of noninvasive ventilation (CPAP) with an oxygen requirement. He had tracheaomalacia and a supraglottic cyst. On discharge a sleep study identified severe obstructive sleep apnea with central apneas, which was managed with overnight noninvasive ventilation. An echocardiogram revealed a structurally normal heart and abdominal ultrasound scan was reported as normal, with no abnormalities of renal system. Cranial MRI and CT scans conducted at 3 months of age revealed bilateral perisylvian polymicrogyria (PMG) more prominent on the right side (Figure 1d, e, respectively) and hypogenesis of the corpus callosum (not shown). An EEG was reported as normal. Metabolic testing including very long chain fatty acids and creatine kinase were normal and congenital infection screening was negative, including CMV testing on the Guthrie card. Blood sent for a postnatal karyotype was reported as normal male karyotype. No clinical or electrical seizures were reported. The occipito‐frontal circumference tracked along the 75th percentile, however, development was significantly developmentally delayed. At 4 months head control was poor and he was unable to fix and follow objects or make purposeful hand movements. At 6 months he was unable to roll or sit, had poor head control but had developed some purposeful hand movements. After a prolonged period in hospital including several intensive care admissions due to recurrent infections the proband was discharged. He re‐presented 3 weeks later with cardiac arrest from which he was resuscitated before spending 4 days in pediatric intensive care where he later died from a RSV positive pneumonitis causing multiorgan failure. He was 10 months old at the time of death.

The second case is a 5‐year‐old male who was born at 37 weeks gestation following a pregnancy that was complicated by concerns about fetal wellbeing due to multiple anomalies detected on antenatal ultrasound, including polycystic disease of the kidney, liver, and pancreas, and intracranial cysts. Amniocentesis showed a normal male karyotype at the 400 band level. Fetal growth and Doppler studies were normal. He was born by spontaneous vaginal delivery and required only facial oxygen at birth. Due to multiple antenatally detected congenital abnormalities he was admitted to the special care baby unit for further investigation. He did not require any respiratory support. He was noted to have head circumference of 36 cm (90th centile), weight 3.07 kg (50th centile), and had facial features consisting of low set ears, cutis aplasia, skin tags on chin, duplicated and medially deviated great toes, anterior bowing of the tibia, clindodactyly of 5th fingers, medially deviated thumbs and hypotonia. He was also found to have undescended testes and multiple skin tags. Cardiac echo on day 1 showed no evidence of any significant structural abnormality. Cranial ultrasound showed moderate ventricular dilation and the corpus callosum was poorly visualized. Abdominal ultrasound confirmed the antenatal findings of cystic disease involving bilaterally enlarged kidneys, liver, pancreas, and spleen. He had small epibulbar dermoids and mildly hypoplastic optic discs. At day 15 of life the proband developed persistent bile stained vomiting and contrast studies revealed a malrotation requiring emergency surgery. He was also noted to be persistently hypertensive requiring him to be discharged on captopril which continues. Very long chain fatty acids and creatinine kinase levels were normal. Conventional chromosome analysis and chromosomal microarray analysis of peripheral blood lymphocytes and skin fibroblasts showed no abnormalities and a male constitution. Interphase FISH on buccal cells using a chromosome 12p13.33 subtelomere probe also showed no abnormalities. However, interphase FISH on skin fibroblasts showed low level mosaicism for trisomy 12p (Figure 1c), and three copies of the 12p13.33 subtelomere probe were found in approximately 14% (27/200) of cells analyzed indicating a duplication, at least partial, of chromosome 12p (data not shown). This finding of mosaic trisomy 12p is consistent with a PKS phenotype (Izumi et al., 2012) as manifested in the proband. Postdischarge from the neonatal unit he remained hypotonic with developmental delay, requiring mostly liquid diet at 4 years of age. MRI performed at age 6 months revealed a global reduction in white matter, prominent Virchow‐Robin spaces, hypogenesis of the corpus callosum, enlarged lateral ventricles and a prominent cisterna magna (Figure 1f–g). He does not exhibit any seizure activity and an EEG was normal.

## 4 DISCUSSION

Pallister‐Killian syndrome (PKS) is characterized cytogenetically by mosaic tetrasomy 12p and the presence of a supernumerary isochromosome composed of the short arms of chromosome 12 (denoted as i(12p)). The PKS phenotype also occurs with trisomy 12p (Izumi et al., 2012). Probands with PKS are frequently stillborn or die in the neonatal period, while diagnosing individuals developing beyond infancy can be challenging because of mosaicism.

Pallister‐Killian syndrome is associated with structural brain abnormalities (Wilkens et al., 2012). To understand phenotypic spectrum of intracranial anomalies associated with PKS, in addition to reporting two new probands, we reviewed the literature for cases of PKS in which an intracranial scan was reported (*n* = 93; see Appendix S1). We observed that 77.41% (72/93) of cases reported a structural brain abnormality. Of these, ventricular abnormalities (45.83%; 33/72), abnormalities of the corpus callosum (25.00%; 18/72) and cerebral atrophy (29.17%; 21/72) were among the most frequently reported. Abnormalities of the corpus callosum represent a more commonly recognized brain malformation which can occur in association with congenital syndromes, and arise from a failure to establish midline brain structures, or failure in the development of cortical neurons or impaired guidance of axons from commissural neurons (Edwards, Sherr, Barkovich, & Richards, 2014). Indeed, genomic copy number variants are prevalent in corpus callosum abnormalities (O'Driscoll, Black, Clayton‐Smith, Sherr, & Dobyns, 2010; Sajan et al., 2013; Sherr et al., 2005). In contrast, macrocephaly (12.5%; 9/72), enlarged or prominent cisterna magna (4.17%; 3/72) and PMG (4.17%; 3/72) were less frequent (Table 1). Nevertheless, PMG is one of the most common malformations of cortical development (MCD), accounting for approximately 20% of all MCDs (Leventer et al., 1999). It is classified as an organizational disorder due to a malformation in late neuronal migration and cortical organization forming multiple small gyri so as to give the appearance macroscopically of an irregular cortical surface. PMG is probably one of the most heterogeneous MCD in terms of associated brain malformations and clinical presentation (Stutterd & Leventer, 2014). PMG is more commonly localized to the perisylvian region seen in approximately 60% of patients studied (Leventer et al., 2010) and the majority of those patients have bilateral perisylvian PMG. PMG is associated with seizures in about 60–80% although they may not present until early adulthood. PMG can also present with significant developmental delay and in the case of perisylvian PMG language delay is a prominent feature (Stutterd & Leventer, 2014).

**Table 1:** Summary of reported structural brain abnormalities in Pallister‐Killian Syndrome (PKS)

Reported feature	Number of reported cases	Frequency (%)	References (see Appendix S1)
Ventricular abnormalities	33	45.83	2, 3, 6, 7, 8, 10, 11, 15, 17, 18, 22, 28, 31, 35, 36, 39, 40, 43, 45, 46, 50, 52, 56, 62, 63, 64
Atrophy	21	29.17	2, 7, 11, 18, 19, 21, 22, 31, 32, 37, 38, 39, 40, 41, 43, 55, 56
Corpus callosum and white matter abnormalities (inc leukomalacia)	18	25.00	6, 8, 13, 18, 22, 24, 25, 27, 38, 40, 42, 46, 57, This study
Macrocephaly	9	12.50	16, 25, 29, 38, 58, 59, 60, 61, 62, This study
Cerebellar structural defects	8	11.11	15, 19, 21, 25, 27, 34, 37, 51
Hydrocephalus, hygroma	5	6.94	4, 23, 34, 36, 55
Others (olfactory bulb hypoplasia, germinal matrix abnormalities, cerebral abnormalities)	4	5.56	19, 51, 57
Polymicrogyria	3	4.17	2, 3, This study
Cisterna magna	3	4.17	32, 48, This study
Pachygyria	2	2.78	22
Microcephaly	1	1.39	27
No adverse features from intracranial scan	21	22.58	5, 6, 12, 14, 22, 26, 30, 41, 44, 47, 49, 53, 54
Total number of intracranial scans	93		

We surveyed over 150 published reports in the literature to identify 93 cases of PKS diagnosis accompanied by intracranial MRI data (see Appendix S1 for cited studies). We cannot rule out detection bias as a confound in our results.

It is reported that approximately 50% of PKS patients exhibit seizures although the time of presentation of seizures varies from infancy to adulthood, and there is no specific seizure type or EEG pattern (Candee, Carey, Krantz, & Filloux, 2012; Giordano et al., 2012; Wilkens et al., 2012). Ostensibly, a combination of PKS and brain abnormality would lead to increased risk of seizure development over time, thereby contributing to significant development delay or intellectual impairment. We observe that while the phenotypic spectrum of PKS is highly variable, the presence or absence of *PMG* or other documented structural brain abnormalities may help prognosticate neurodevelopmental outcome and risk of seizures as the child ages. Hence, our findings suggest that neuroimaging could be informative even in cases of PKS where seizures are not evident.

In PKS, mosaic tetrasomy 12p could lead to aberrant gene expression within cells of the developing fetal brain. We surveyed the gene expression database FANTOM5 (Consortium et al., 2014) and identified at least 63 out of 341 candidate genes (18.47%) on 12p that are enriched in tissues of the nervous system, suggesting their direct roles in neurodevelopment and disease (Fig. S1a and Table S1 for details). We charted the developmental expression profile for these 63 genes, as well as their regional patterns in human fetal and adult brain tissues (Fig. S1b and Table S2). Interestingly, genes such as *ERC1*,* CCND2*,* APOLD1*,* GRIN2B*,* PTPRO*,* EPS8*,* SOX5,* and *BICD1* were enriched in fetal brain tissues, with lower expression levels in adult brain tissues. On the other hand, genes such as *GABARAPL1* and *MANSC1* were relatively low in expression in fetal brain, but levels were elevated in adult tissue. In addition, several genes such as *GSG1* and *PDE6H* showed region‐specific (pineal gland) expression, while others such as *C12ORF57* and *PRR4* were lowly expressed in the brain.

To understand how disruptions to these 12p genes might underlie neuropathological features in PKS, we mapped minimal critical regions for reported cases with neuropathological features highlighted in Table 1 (Fig. S2 and Table S3). We observed a critical minimal region spanning *12p13.33* to *12p13.2* for atrophy comprising neuronal genes *IQSEC3, SLC6A12, RAD52, ERC1, FBXL14, ADIPO2, FKBP2, NRIP2, TULP3, CCND2, C12ORF57, OLR1, GABARAPL1, PRR4* (Fig. S2a). We also identified a minimal critical region for corpus callosum abnormalities spanning *12p13.33* through to *12p12.3*, encompassing *LRP6, MANSC1, DUSP16, CREBL2, GPR19, CDKN1B, APOLD1, KIAA1467, GSG1, GRIN2B, WBP11, C12OR60, PDE6H, RERG, PTPRO, EPS8, STRAP, LMO3* (Fig. S2b). The overlapping critical regions could suggest common genetic mechanisms for atrophy and callosal defects mediated by increased *12p13.33*‐*12p13.2* gene dosage, with additional genes from *12p13.2‐12p12.3* unique to the corpus callosum. In contrast, we identified a minimal critical region encompassing *12p12.3* to *12p11.22* for macrocephaly which was distinct to the two previous traits, and implicates a separate cluster of *12p* genes (*RERGL, PLEKHA5, AEBP2, SLCO1C1, SLCO1C2, LDHB, ABCC9, CMAS, ST8SIA1, ETNK1, SOX5, BCAT1, CASC1, LYRM5, RASSF8, SSPN, ITPRN2, FGFR1OP2, MED21, CCDC91, FAR2, ERGIC2, TMTC1*) (Fig. S2c). Minimal critical regions could not be established for the remaining traits highlighted in our study (Table 1) (data not shown). From these observations, our study extends the findings of Izumi et al. (2012) as well as Wilkens et al. (2012) in defining distinct 12p loci and their constituent genes to neurological traits in PKS. For example, disruptions to chromosome 12p genes implicated in atrophy and callosal defects in PKS such as *CCND2* are associated with megalencephaly‐polymicrogyria‐polydactyly‐hydrocephalus syndrome (OMIM# 123833) (Mirzaa et al., 2014), while *C12ORF57* is implicated in Temtamy Syndrome (OMIM# 615140); a condition characterized by mental retardation, multiple congenital anomaly including abnormalities of the corpus callosum (Akizu et al., 2013), as well as a syndromic form of Colobomatous Microphthalmia associated with profound global developmental delay, intractable seizures, and corpus callosum abnormalities (Zahrani, Aldahmesh, Alshammari, Al‐Hazzaa, & Alkuraya, 2013). As a further example, mutations to *GRIN2B* (a gene which maps to an exclusive minimal critical region for corpus callosum abnormalities) are associated with a neurodevelopmental disorders characterized by a spectrum of malformations, including hypoplasia of the corpus callosum, seizures and MCD (MDR6; OMIM# 613970). In the case of genes within the 12p critical region implicated in macrocephaly in PKS, mutations to *ABCC9* which encodes the sulfonylurea receptor (SUR) that forms ATP‐sensitive potassium channels (K_ATP_) channels are associated with Cantú syndrome, a rare syndromic overgrowth condition which presents with macrocephaly (OMIM# 239850). A better understanding of the functions for 12p genes within these critical regions will facilitate our understanding of their direct roles in brain development, and how elevations in 12p gene dosage are causative for neuropathological features in PKS.

It is notable that PKS can arise as a consequence of cytogenetic mosaicism, and this may have contributed to the paucity of previous reports of the association of PKS with congenital brain abnormalities. Specifically, mosaic tetrasomy and trisomy 12p may not be detected by conventional chromosome analysis or microarray analysis of peripheral blood lymphocytes, and other cell types (e.g., skin fibroblasts) and techniques better suited to detection of low level mosaicism such as interphase FISH may be required to confirm the diagnosis, as was the case with Proband 2. Interestingly, in addition to the neurological traits for Proband 2, this child presented with evidence of cystic disease involving liver, pancreas and spleen; as well as small epibulbar dermoids and hypoplastic optic discs which are unusual for PKS. The identification of further subjects with very low frequency of 12p aneuploidy will establish a stronger association between these unusual features and PKS.

## AUTHOR CONTRIBUTIONS

CP and GB contributed clinical details and data, CGY and HAR contributed data, HW provided clinical details and patient consent; Clinical genetics provided by DR; Diagnostic genomics provided by KW, SS, JP and JB, MRI imaging data from PS; CP wrote the manuscript with JIH.

## CONFLICT OF INTEREST

None declared.

## Supporting information

* *

* *
