# Supplementary figures and images for: A review of structural brain abnormalities in Pallister‐Killian syndrome
Source: Mol Genet Genomic Med. 2017 Dec 9;6(1):92–8. doi: 10.1002/mgg3.351 (PMC5823685; doi:10.1002/mgg3.351)

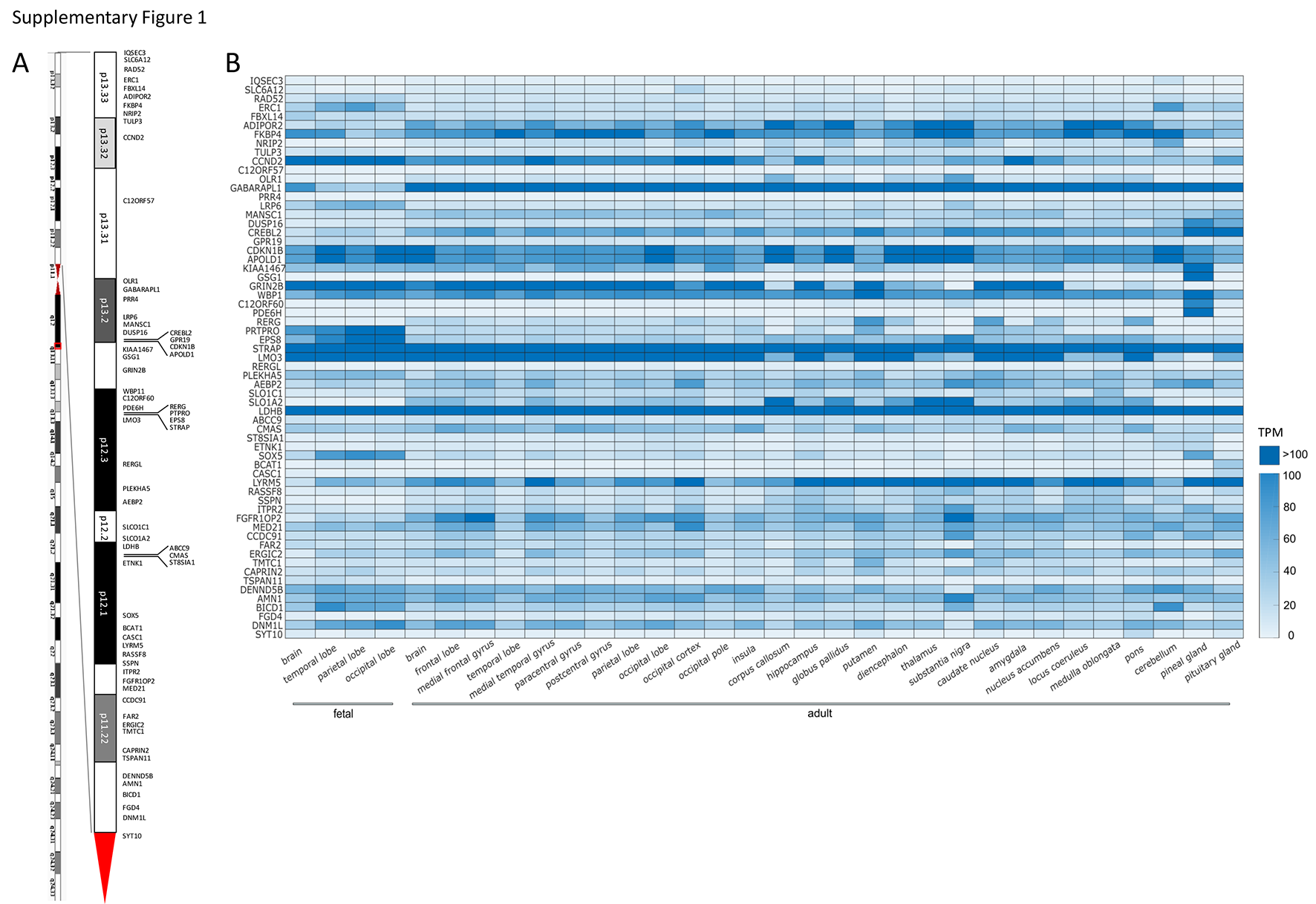

Supplement: Supplementary file 1 [file MGG3-6-92-s001.tif]

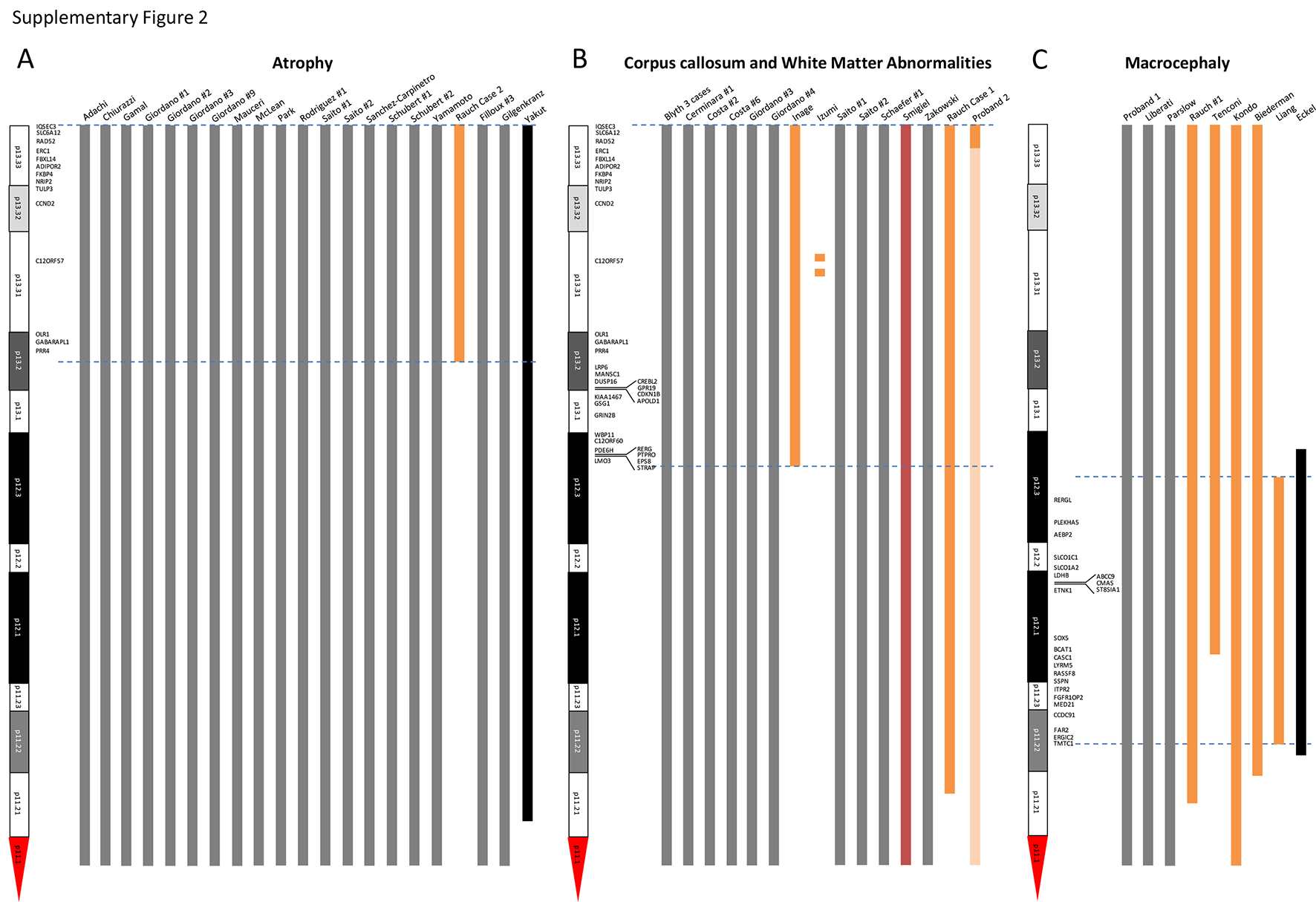

Supplement: Supplementary file 2 [file MGG3-6-92-s002.tif]
