# Supplementary material for: A review of structural brain abnormalities in Pallister‐Killian syndrome
Source: Mol Genet Genomic Med. 2017 Dec 9;6(1):92–8. doi: 10.1002/mgg3.351 (PMC5823685; doi:10.1002/mgg3.351)
Supplement: Supplementary file 6 [file MGG3-6-92-s006.docx]

Supplementary References (related to Table 1):

1 Abad, D. E. *et al.* Pallister-Killian syndrome presenting with a complex congenital heart defect and increased nuchal translucency. *J Ultrasound Med* **25**, 1475-1480 (2006).

2 Adachi, M. *et al.* Pallister-Mosaic syndrome and neuronal migration disorder. *Brain Dev* **25**, 357-361 (2003).

3 Baglaj, M., King, J. & Carachi, R. Pallister-Killian syndrome: a report of 2 cases and review of its surgical aspects. *J Pediatr Surg* **43**, 1218-1221, doi:10.1016/j.jpedsurg.2008.03.030 (2008).

4 Bernert, J. *et al.* Prenatal diagnosis of the Pallister-Killian mosaic aneuploidy syndrome by CVS. *Am J Med Genet* **42**, 747-750, doi:10.1002/ajmg.1320420525 (1992).

5 Bielanska, M. M., Khalifa, M. M. & Duncan, A. M. Pallister-Killian syndrome: a mild case diagnosed by fluorescence in situ hybridization. Review of the literature and expansion of the phenotype. *Am J Med Genet* **65**, 104-108, doi:10.1002/(SICI)1096-8628(19961016)65:2<104::AID-AJMG4>3.0.CO;2-S (1996).

6 Blyth, M. *et al.* Pallister-Killian syndrome: a study of 22 British patients. *J Med Genet* **52**, 454-464, doi:10.1136/jmedgenet-2014-102877 (2015).

7 Boyle, A. H., Kulkarni, R., Smoleniec, J. S., Davies, T. & McDermott, A. Prenatal diagnosis of Pallister-Killian syndrome by chorionic villus sampling--its diagnostic problems. *Prenat Diagn* **13**, 1160-1161 (1993).

8 Cerminara, C. *et al.* Late-onset epileptic spasms in children with Pallister-Killian syndrome: a report of two new cases and review of the electroclinical aspects. *J Child Neurol* **25**, 238-245, doi:10.1177/0883073809336933 (2010).

9 Chen, C. P. *et al.* Mosaic tetrasomy 12p with discrepancy between fetal tissues and extraembryonic tissues: molecular analysis and possible mechanism of formation. *Taiwan J Obstet Gynecol* **49**, 235-238, doi:10.1016/S1028-4559(10)60053-8 (2010).

10 Chiesa, J. *et al.* Pallister-Killian syndrome [i(12p)]: first pre-natal diagnosis using cordocentesis in the second trimester confirmed by in situ hybridization. *Clin Genet* **54**, 294-302 (1998).

11 Chiurazzi, P. *et al.* Assisted reproductive technology and congenital overgrowth: some speculations on a case of Pallister-Killian syndrome. *Am J Med Genet A* **130A**, 315-316, doi:10.1002/ajmg.a.30300 (2004).

12 Choo, S., Teo, S. H., Tan, M., Yong, M. H. & Ho, L. Y. Tissue-limited mosaicism in Pallister-Killian syndrome -- a case in point. *J Perinatol* **22**, 420-423, doi:10.1038/sj.jp.7210712 (2002).

13 Costa, L. S. *et al.* Cytogenomic delineation and clinical follow-up of 10 Brazilian patients with Pallister-Killian syndrome. *Mol Cytogenet* **8**, 43, doi:10.1186/s13039-015-0142-7 (2015).

14 Dong, L., Falk, R. E., Williams, J., 3rd, Kohan, M. & Schreck, R. R. Tetrasomy 12p--unusual presentation in CVS. *Prenat Diagn* **23**, 101-103, doi:10.1002/pd.538 (2003).

15 Doray, B. *et al.* Pallister-Killian syndrome: difficulties of prenatal diagnosis. *Prenat Diagn* **22**, 470-477, doi:10.1002/pd.342 (2002).

16 Eckel, H. *et al.* Intrachromosomal triplication 12p11.22-p12.3 and gonadal mosaicism of partial tetrasomy 12p. *Am J Med Genet A* **140**, 1219-1222, doi:10.1002/ajmg.a.31237 (2006).

17 el-Naggar, M. & Hawthorne, M. Pallister-Killian syndrome: an unusual presentation. *J Laryngol Otol* **108**, 669-670 (1994).

18 Filloux, F. M., Carey, J. C., Krantz, I. D., Ekstrand, J. J. & Candee, M. S. Occurrence and clinical features of epileptic and non-epileptic paroxysmal events in five children with Pallister-Killian syndrome. *Eur J Med Genet* **55**, 367-373, doi:10.1016/j.ejmg.2012.01.006 (2012).

19 Gamal, S. M. *et al.* Cytogenetic study of a severe case of Pallister-Killian syndrome using fluorescence in situ hybridization. *Jpn J Hum Genet* **39**, 259-267, doi:10.1007/BF01876847 (1994).

20 Gerdes, A. M. *et al.* Pallister-Killian syndrome: Multiband FISH of tetrasomy 12p. *Pediatr Dermatol* **23**, 378-381, doi:10.1111/j.1525-1470.2006.00250.x (2006).

21 Gilgenkrantz, S. *et al.* Mosaic tetrasomy 12p. *Clin Genet* **28**, 495-502 (1985).

22 Giordano, L. *et al.* Seizures and EEG patterns in Pallister-Killian syndrome: 13 new Italian patients. *Eur J Paediatr Neurol* **16**, 636-641, doi:10.1016/j.ejpn.2012.03.003 (2012).

23 Hunter, A. G., Clifford, B. & Cox, D. M. The characteristic physiognomy and tissue specific karyotype distribution in the Pallister-Killian syndrome. *Clin Genet* **28**, 47-53 (1985).

24 Inage, E. *et al.* Phenotypic overlapping of trisomy 12p and Pallister-Killian syndrome. *Eur J Med Genet* **53**, 159-161, doi:10.1016/j.ejmg.2010.02.009 (2010).

25 Izumi, K. *et al.* Duplication 12p and Pallister-Killian syndrome: a case report and review of the literature toward defining a Pallister-Killian syndrome minimal critical region. *Am J Med Genet A* **158A**, 3033-3045, doi:10.1002/ajmg.a.35500 (2012).

26 Kunz, J., Schoner, K., Stein, W., Rehder, H. & Fritz, B. Tetrasomy 12p (Pallister-Killian syndrome): difficulties in prenatal diagnosis. *Arch Gynecol Obstet* **280**, 1049-1053, doi:10.1007/s00404-009-1059-3 (2009).

27 Lalatta, F., Salmona, S., Fogliani, R., Rizzuti, T. & Nicolini, U. Prenatal diagnosis of genetic syndromes may be facilitated by serendipitous findings at fetal blood sampling. *Prenat Diagn* **18**, 834-837 (1998).

28 Leube, B., Majewski, F., Gebauer, J. & Royer-Pokora, B. Clinical, cytogenetic, and molecular observations in a patient with Pallister-Killian-syndrome with an unusual karyotype. *Am J Med Genet A* **123A**, 296-300, doi:10.1002/ajmg.a.20339 (2003).

29 Liberati, M. *et al.* Fetal facial profile in Pallister-Killian syndrome. *Fetal Diagn Ther* **23**, 15-17, doi:10.1159/000109220 (2008).

30 Los, F. J. *et al.* Prenatal diagnosis of mosaic tetrasomy 12p/trisomy 12p by fluorescent in situ hybridization in amniotic fluid cells: a case report of Pallister-Killian syndrome. *Prenat Diagn* **15**, 1155-1159 (1995).

31 Mauceri, L., Sorge, G., Incorpora, G. & Pavone, L. Pallister-Killian syndrome: case report with pineal tumor. *Am J Med Genet* **95**, 75-78 (2000).

32 McLean, S. *et al.* Prenatal diagnosis of Pallister-Killian syndrome: resolution of cytogenetic ambiguity by use of fluorescent in situ hybridization. *Prenat Diagn* **12**, 985-991 (1992).

33 McPherson, E. W., Ketterer, D. M. & Salsburey, D. J. Pallister-Killian and Fryns syndromes: nosology. *Am J Med Genet* **47**, 241-245, doi:10.1002/ajmg.1320470219 (1993).

34 Mourali, M. *et al.* First trimester diagnosis of Pallister-Killian syndrome in a fetus with suggestive abnormalities. *Tunis Med* **88**, 666-669 (2010).

35 Narahara, K. *et al.* Pallister-Killian syndrome: cytogenetic and biochemical studies. *Jinrui Idengaku Zasshi* **33**, 339-347 (1988).

36 Paladini, D. *et al.* Prospective ultrasound diagnosis of Pallister-Killian syndrome in the second trimester of pregnancy: the importance of the fetal facial profile. *Prenat Diagn* **20**, 996-998 (2000).

37 Park, I. Y. *et al.* Prenatal diagnosis of Pallister-Killian syndrome associated with pulmonary stenosis and right ventricular dilatation. *Korean J Lab Med* **29**, 366-370, doi:10.3343/kjlm.2009.29.4.366 (2009).

38 Rauch, A., Trautmann, U. & Pfeiffer, R. A. Clinical and molecular cytogenetic observations in three cases of "trisomy 12p syndrome". *Am J Med Genet* **63**, 243-249, doi:10.1002/(SICI)1096-8628(19960503)63:1<243::AID-AJMG42>3.0.CO;2-L (1996).

39 Rodriguez, J. I., Garcia, I., Alvarez, J., Delicado, A. & Palacios, J. Lethal Pallister-Killian syndrome: phenotypic similarity with Fryns syndrome. *Am J Med Genet* **53**, 176-181, doi:10.1002/ajmg.1320530211 (1994).

40 Saito, Y. *et al.* Brain MRI findings of older patients with Pallister-Killian syndrome. *Brain Dev* **28**, 34-38, doi:10.1016/j.braindev.2005.04.004 (2006).

41 Sanchez-Carpintero, R. *et al.* Pallister-Killian syndrome: an unusual cause of epileptic spasms. *Dev Med Child Neurol* **47**, 776-779, doi:10.1017/S0012162205001623 (2005).

42 Schaefer, G. B., Jochar, A., Muneer, R. & Sanger, W. G. Clinical variability of tetrasomy 12p. *Clin Genet* **51**, 102-108 (1997).

43 Schubert, R., Viersbach, R., Eggermann, T., Hansmann, M. & Schwanitz, G. Report of two new cases of Pallister-Killian syndrome confirmed by FISH: tissue-specific mosaicism and loss of i(12p) by in vitro selection. *Am J Med Genet* **72**, 106-110 (1997).

44 Shah, K. *et al.* An Indian boy with additional features in Pallister-Killian syndrome. *Indian J Pediatr* **79**, 1238-1240, doi:10.1007/s12098-011-0585-8 (2012).

45 Shen, J. D. *et al.* Pallister-Killian syndrome: meiosis II non-disjunction may be the first step in the formation of isochromosome 12p. *Chin Med J (Engl)* **123**, 3482-3485 (2010).

46 Smigiel, R. *et al.* The Pallister-Killian syndrome in a child with rare karyotype--a diagnostic problem. *Eur J Pediatr* **167**, 1063-1065, doi:10.1007/s00431-007-0608-7 (2008).

47 Soukup, S. & Neidich, K. Prenatal diagnosis of Pallister-Killian syndrome. *Am J Med Genet* **35**, 526-528, doi:10.1002/ajmg.1320350417 (1990).

48 Srinivasan, A. & Wright, D. Pallister-Killian syndrome. *Am J Case Rep* **15**, 194-198, doi:10.12659/AJCR.890614 (2014).

49 Stalker, H. J., Gray, B. A., Bent-Williams, A. & Zori, R. T. High cognitive functioning and behavioral phenotype in Pallister-Killian syndrome. *Am J Med Genet A* **140**, 1950-1954, doi:10.1002/ajmg.a.31403 (2006).

50 Sukarova-Angelovska, E., Kocova, M., Ilieva, G., Angelkova, N. & Kochova, E. Rare case of Killian-Pallister syndrome associated with idiopathic short stature detected with fluorescent in situ hybridization on buccal smear. *Mol Cytogenet* **9**, 38, doi:10.1186/s13039-016-0239-7 (2016).

51 Turleau, C. *et al.* Parental origin and mechanisms of formation of three cases of 12p tetrasomy. *Clin Genet* **50**, 41-46 (1996).

52 Velagaleti, G. V. *et al.* A rapid and noninvasive method for detecting tissue-limited mosaicism: detection of i(12)(p10) in buccal smear from a child with Pallister-Killian syndrome. *Genet Test* **7**, 219-223, doi:10.1089/109065703322537232 (2003).

53 Vermeesch, J. R. *et al.* Tetrasomy 12pter-12p13.31 in a girl with partial Pallister-Killian syndrome phenotype. *Eur J Med Genet* **48**, 319-327, doi:10.1016/j.ejmg.2005.04.018 (2005).

54 Vogel, I., Lyngbye, T., Nielsen, A., Pedersen, S. & Hertz, J. M. Pallister-Killian syndrome in a girl with mild developmental delay and mosaicism for hexasomy 12p. *Am J Med Genet A* **149A**, 510-514, doi:10.1002/ajmg.a.32681 (2009).

55 Yakut, S. *et al.* Mosaic Intrachromosomal Triplication of (12)(p11.2p13) in a Patient with Pallister-Killian Syndrome. *Balkan J Med Genet* **15**, 61-64, doi:10.2478/v10034-012-0010-2 (2012).

56 Yamamoto, H., Fukuda, M., Murakami, H., Kamiyama, N. & Miyamoto, Y. A case of Pallister-Killian syndrome associated with West syndrome. *Pediatr Neurol* **37**, 226-228, doi:10.1016/j.pediatrneurol.2007.05.001 (2007).

57 Zakowski, M. F., Wright, Y. & Ricci, A., Jr. Pericardial agenesis and focal aplasia cutis in tetrasomy 12p (Pallister-Killian syndrome). *Am J Med Genet* **42**, 323-325, doi:10.1002/ajmg.1320420313 (1992).

58 Biederman, B., Bowen, P., Robertson, C. & Schiff, D. Partial trisomy 12p due to t(12;21)pat translocation. *Hum Genet* **36**, 35-41 (1977).

59 Kondo, I., Hamaguchi, H. & Haneda, T. Trisomy 12p syndrome: de novo occurrence of mosaic trisomy 12p in a mentally retarded boy. *Hum Genet* **46**, 135-140 (1979).

60 Liang, D. et al. A father and son with mental retardation, a characteristic face, inv(12), and insertion trisomy 12p12.3-p11.2. *Am J Med Genet A* **140**, 238-244, doi:10.1002/ajmg.a.31077 (2006).

61 Parslow, M., Chambers, D., Drummond, M. & Hunter, W. Two cases of trisomy 12p due to rcpt (12;21)(p11;p11) inherited through three generations. *Hum Genet* **47**, 253-260 (1979).

62 Tenconi, R., Piovan, E., Preto, A., Magnabosco, R. & Baccichetti, C. Syndrome +12p. Case report and review. *Hum Genet* **39**, 97-101 (1977).
